# Fine tuned personalized machine learning models to detect insomnia risk based on data from a smart bed platform

**DOI:** 10.3389/fneur.2024.1303978

**Published:** 2024-02-14

**Authors:** Trevor Winger, Vidhya Chellamuthu, Dmytro Guzenko, Mark Aloia, Shawn Barr, Susan DeFranco, Brandon Gorski, Faisal Mushtaq, Gary Garcia-Molina

**Affiliations:** ^1^Sleep Number Labs, Sleep Number, San Jose, CA, United States; ^2^Department of Computer Science and Engineering, University of Minnesota, Minneapolis, MN, United States; ^3^GlobalLogic, Kyiv, Ukraine; ^4^Sleep Number Corporation, Minneapolis, MN, United States; ^5^National Jewish Health, Denver, CO, United States; ^6^Department of Psychiatry, University of Wisconsin-Madison, Madison, WI, United States

**Keywords:** insomnia risk, personalized machine learning, incremental learning, fine tuning, passive-aggressive learning

## Abstract

**Introduction:**

Insomnia causes serious adverse health effects and is estimated to affect 10–30% of the worldwide population. This study leverages personalized fine-tuned machine learning algorithms to detect insomnia risk based on questionnaire and longitudinal objective sleep data collected by a smart bed platform.

**Methods:**

Users of the Sleep Number smart bed were invited to participate in an IRB approved study which required them to respond to four questionnaires (which included the Insomnia Severity Index; ISI) administered 6 weeks apart from each other in the period from November 2021 to March 2022. For 1,489 participants who completed at least 3 questionnaires, objective data (which includes sleep/wake and cardio-respiratory metrics) collected by the platform were queried for analysis. An incremental, passive-aggressive machine learning model was used to detect insomnia risk which was defined by the ISI exceeding a given threshold. Three ISI thresholds (8, 10, and 15) were considered. The incremental model is advantageous because it allows personalized fine-tuning by adding individual training data to a generic model.

**Results:**

The generic model, without personalizing, resulted in an area under the receiving-operating curve (AUC) of about 0.5 for each ISI threshold. The personalized fine-tuning with the data of just five sleep sessions from the individual for whom the model is being personalized resulted in AUCs exceeding 0.8 for all ISI thresholds. Interestingly, no further AUC enhancements resulted by adding personalized data exceeding ten sessions.

**Discussion:**

These are encouraging results motivating further investigation into the application of personalized fine tuning machine learning to detect insomnia risk based on longitudinal sleep data and the extension of this paradigm to sleep medicine.

## 1 Introduction

Insomnia is a highly prevalent sleep disorder, affecting 10–30% of the general population (1), which is characterized by difficulty with sleep initiation, weakened sleep maintenance, and/or waking-up too early (1). Insomnia can cause significant distress for those who experience symptoms and has been bidirectionally associated with adverse health consequences such as heart disease, elevated blood pressure, neurological conditions, chronic pain, gastrointestinal problems (2), depression, and anxiety (2). Insomnia can be intermittent, i.e., it is interspersed with occasional good rebound nights. This can give the patient a false sense of remission which may cause low reporting of insomnia to the healthcare system.

Despite its high prevalence, insomnia is underrecognized, underdiagnosed, and undertreated (3). Latest progress in machine learning and the use of consumer sleep technologies may be helpful to alleviate underdiagnosis of multiple sleep disorders including insomnia.

Previous categorization of insomnia into primary and secondary (or comorbid) insomnia has been abandoned (4). Instead, the phenotypes of sleep onset insomnia (difficulty falling asleep), sleep maintenance insomnia (difficulty staying asleep), early morning awakening insomnia, and a combination of those are considered. Another categorization considers the duration of insomnia symptoms and identifies three categories acute (shorter than a month), subacute (1 to 3 months), and chronic insomnia (longer than 3 months) (5).

The Insomnia Severity Index (ISI) is the only instrument currently in use that allows for severity classification depending on a numerical score (6). The ISI has not yet been validated to identify a specific insomnia phenotype, but the identification of insomnia risk can be defined as the ISI exceeding a threshold (7).

Table 1 summarizes some of the approaches in the state-of-the-art to detect insomnia. Park et al. (8) used actigraphy and demographic data with neural-net based clustering techniques to identify five clusters associated with distinct Insomnia endotypes. Rodríguez-Morilla et al. (9) used physiological and body position data along with environmental light exposure to predict primary insomnia using a decision tree model. MRI data were used by Spiegelhalder et al. (10) and Li et al. (11), with a Support Vector Machine classifier. Andrillon et al. (12) leveraged Polysomnography (PSG) to detect chronic insomnia, achieving a high Cohen's κ score of 0.87 using a CaRET (Classification and Regression Training) model. Shahin et al. (13) used EEG data and Support Vector Machines, achieving a high F1 score (0.88) in predicting primary insomnia.

**Table 1:** State-of-the-art of machine learning algorithms applied to insomnia.

**Study**	**Data source**	**Insomnia source**	**ML technique**	**Performance metric**	**Prediction task**
Park et al. (8)	Actigraphy from wearable, demographic data	ISI questionnaire	Neural Net Based Unsupervised Clustering	Found 5 new insomnia-activity clusters achieving average silhouette score ≥0.4 to prove significance	Clustering for Activity-Insomnia endotypes
Rodríguez-Morilla et al. (9)	Skin temperature, motor activity, and body position from several devices	Primary insomnia diagnosis	Decision tree	*Acc*. = 0.884, *Se*. = 0.885, *Sp*. = 0.714, *AUC* = 0.897, *F*1 = 0.921	Binary Classification of Primary Insomnia Diagnosis
Spiegelhalder et al. (10)	MRI	Primary insomnia diagnosis	Support vector machine	Altered brain function related to insomnia appears to not have a substantial effect on brain morphometry on a macroscopic level	Classification of primary insomnia diagnosis (PI used for grouping brain structures)
Li et al. (11)	MRI	Primary insomnia diagnosis	Support vector machine	*Acc*. = 0.815, *Se*. = 0.849, *Sp*. = 0.791, *AUC* = 0.83	Classification of PI diagnosis (Binary)
Andrillon et al. (12)	PSG	Diagnosed with CI according to the ICSD3 criteria	Classification and regression training (CaRET)	*Cohen*′*s κ* = 0.87	Predicting good sleepers compares to CI sleepers
Shahin et al. (13)	EEG	Primary insomnia diagnosis	Support vector machine	*F*1 = 0.88, *Se*. = 0.84, *Sp*. = 0.91	Classification of primary insomnia diagnosis (Binary)

Among the various consumer sleep technologies, it is reasonable to assume that “nearables” which do not require the sleeper to wear any monitor (14) have the potential to reflect real-life longitudinal sleep trends enabling the detection of sleep disturbances and early interventions. This study leveraged the capabilities of a smart bed platform to unobtrusively collect longitudinal objective sleep data and questionnaire responses from a large cohort of individuals to build personalized machine learning models to detect insomnia risk.

## 2 Materials and methods

### 2.1 Questionnaire procedure

Individuals enrolled in the study are owners of a Sleep Number smart bed who consented to participate in an IRB approved study which consisted in responding to four electronically delivered questionnaires and allowing the use of objective sleep data collected by the smart bed platform. The four questionnaires were presented to the enrolled participants on November 22, 2021, January 3, 2022, February 14, 2022, and March 28, 2022 respectively. Each questionnaire was active for two weeks. The objective sleep data were collected between October 21, 2021 and March 31, 2022.

Demographic information including age and gender were collected in the first questionnaire. Each questionnaire was composed of five validated instruments, insomnia severity index (6), Epworth sleepiness scale (ESS) (15), reduced morningness-eveningness questionnaire (16), general anxiety disorder GAD-7 (17), and the patient health questionnaire PHQ-8 (18). The ISI and ESS were administered under a utilization license provided by Mapi Research Trust.

To quantify insomnia risk, we focused on the ISI which is a seven-question instrument designed to assess the severity of both daytime and nighttime components of insomnia. The responses to the 7 ISI questions in a scale from 0 to 4, are added up to obtain a total score which indicates, no clinical significant insomnia if the score is lower than 8, subthreshold insomnia if the score is between 8 and 14, clinical insomnia if the score is between 15 and 21, and severe clinical insomnia if the score is between 22 and 28 (6). For convenience, the total ISI score is simply referred to as ISI in the rest of the paper.

### 2.2 Sleep session data

Sleep session data are collected on a daily basis by the smart bed using the technology and algorithms described in Siyahjani et al. (19). The smart bed, validated against polysomnography (19), uses a pressure sensor to capture high-resolution full body ballistocardiography to accurately measure breathing rate, heart rate and movements to derive *session data*. The smart bed uses a pressure sensor for each sleeper on the bed.

Sleep session data include (see Table 2) the session duration which corresponds to time in bed, the number of bed exits, sleep duration, duration of restful sleep (which was detected based on the level of motion), time to fall asleep (TTFA) once the participant entered the bed, the percentage of time with high (above a given threshold) level motion, sleep quality score, sleep debt which is the difference (if positive) between and individual's sleep duration goal minus their actual sleep duration, sleep regularity which characterizes the probability of an individual of being awake or asleep at any given two points in time separated 24 h apart [using an adaptation of the procedure presented in Lunsford-Avery et al. (20)], and mean cardiorespiratory metrics such as respiratory rate, heart rate, and heart rate variability. The feature vector used to train the machine learning model has 14 components listed in Table 2 (see also **Figure 3**).

**Table 2:** Features and description.

**Feature**	**Description**
Age	Age in years of each participant
Bed exits	Number of times a participant entered and exited the bed during the sleep session. Exits and enters are considered part of the same sleep session if they occur within a 2-h threshold
Breathing rate	The average respiration rate during the sleep session
Gender	Encoded the gender of each participant as a binary variable
Heart rate	The average heart rate during the sleep session
Heart rate variability	Standard deviation of the inter-beat interval across the entire sleep session
Percent motion during sleep session	Percentage of time spent moving during the recorded sleep session
Restful sleep duration	Amount of time in bed considered restful (where motion level is below a pre-established threshold)
Session duration	Total time spent in bed during the recorded session
Sleep debt	Participant sleep goal minus sleep duration
Sleep duration	Time spent in asleep during the recorded session
Sleep IQ score	A proprietary metric used by Sleep Number to measure the quality of sleep for an individual
Sleep regularity index (SRI)	The probability of a person being asleep or awake at any given two points 24 h apart (20)
Time to fall asleep	Amount of time it takes for an individual to fall asleep once they have entered the bed

### 2.3 Data inclusion procedure

On a daily basis, the smart bed consolidated sleep sessions whose end and begin times were not separated by more than two hours. Sleep sessions separated by more than two hours were considered as individual sessions. For each day, only the longest sleep session was kept for analysis.

**Figure 1:**
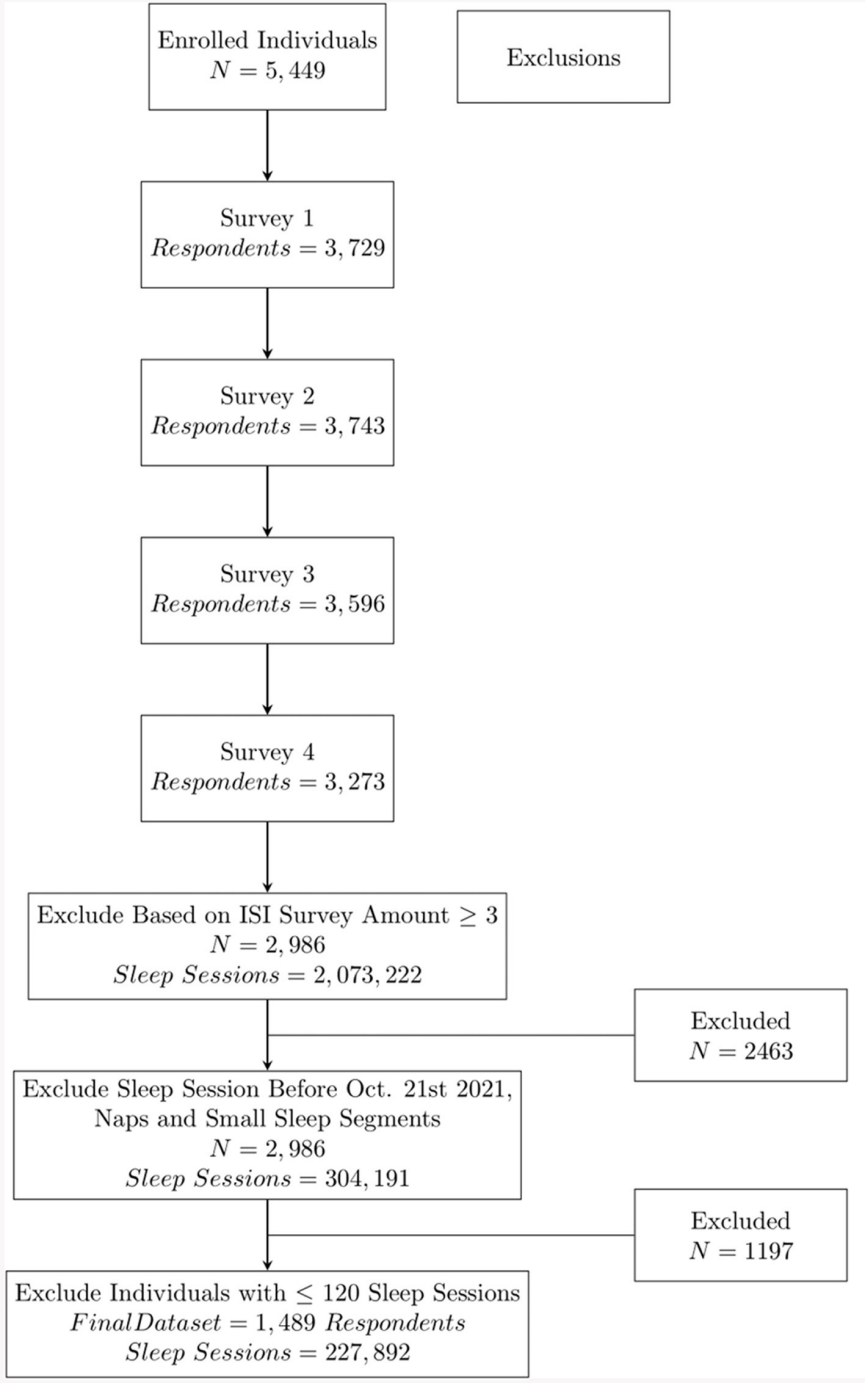
Data inclusion procedure.

Starting from 5,444 enrolled participants, the number of respondents to questionnaires 1 to 4 were 3,729, 3,743, 3,596, and 3,273 respectively. The number of participants that responded to at least three surveys was 2,986. The final dataset for analysis consisted of the data from 1,489 participants [mean age 51.72 (SD: 12.77) years-old; 669 Men and 811 Women] who had at least 120 sessions in the period from October 21, 2021 to March 31, 2022. This process is illustrated in Figure 1.

### 2.4 Insomnia risk quantification and labeling of each sleep session

To detect insomnia risk, three thresholds on the ISI (8, 10, and 15) were considered. As mentioned in Section 2.1, the thresholds 8 and 15 distinguish no insomnia vs. any level of insomnia and non-severe insomnia vs. severe insomnia respectively. The ISI threshold of 10 was used by Oh et al. (7) to quantify insomnia risk.

**Figure 2:**
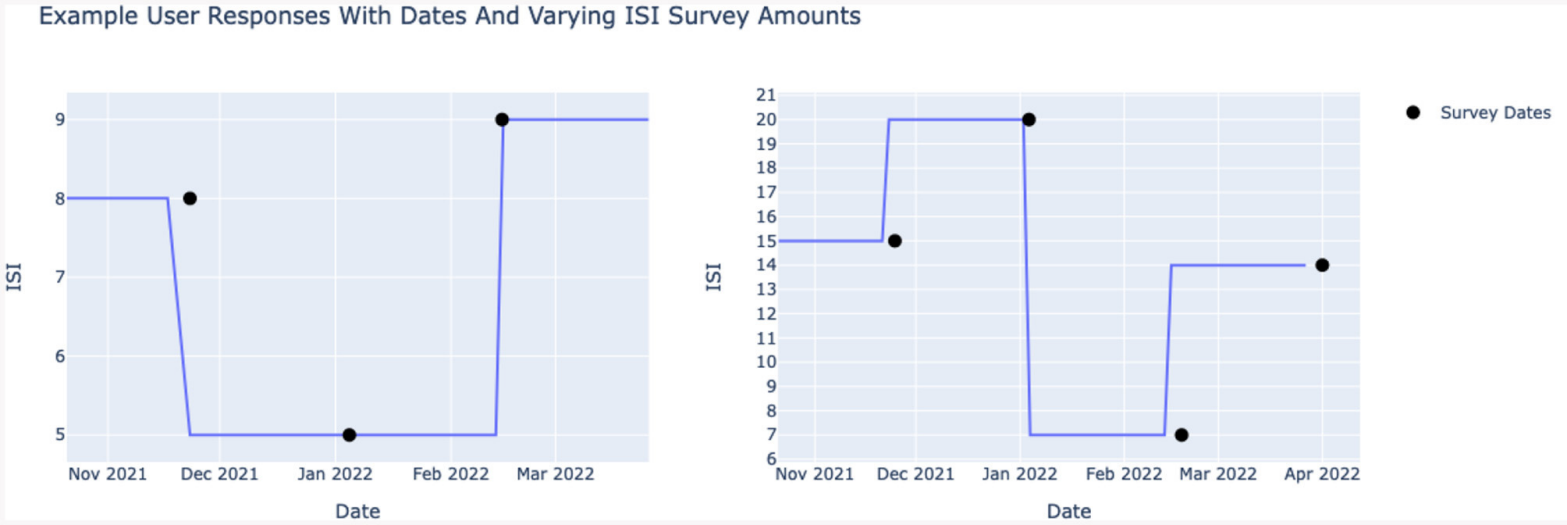
Example of respondent ISI score assignment to sleep sessions.

Each sleep session had an ISI value assigned according to the following criteria (see also Figure 2). For each session before the second questionnaire, the ISI is that of the first questionnaire. If the response to the first questionnaire is missing, then all sessions before the second session have the ISI value of the second questionnaire. For each sleep session after the last questionnaire answered by the participant, the score is the ISI of the last questionnaire. In-between, the sleep sessions between the n-th questionnaire and the (n+1)-th questionnaire have the ISI corresponding to that of the n-th questionnaire.

### 2.5 Personalized fine tuning

In machine learning, the idea of improving a model by transferring information from a related domain is referred to as transfer learning (21). A related concept is that of fine tuning where a generic model is incrementally trained to optimally perform in specific scenarios. The incremental training uses a small amount of training data from the targeted specific scenario.

**Figure 3:**
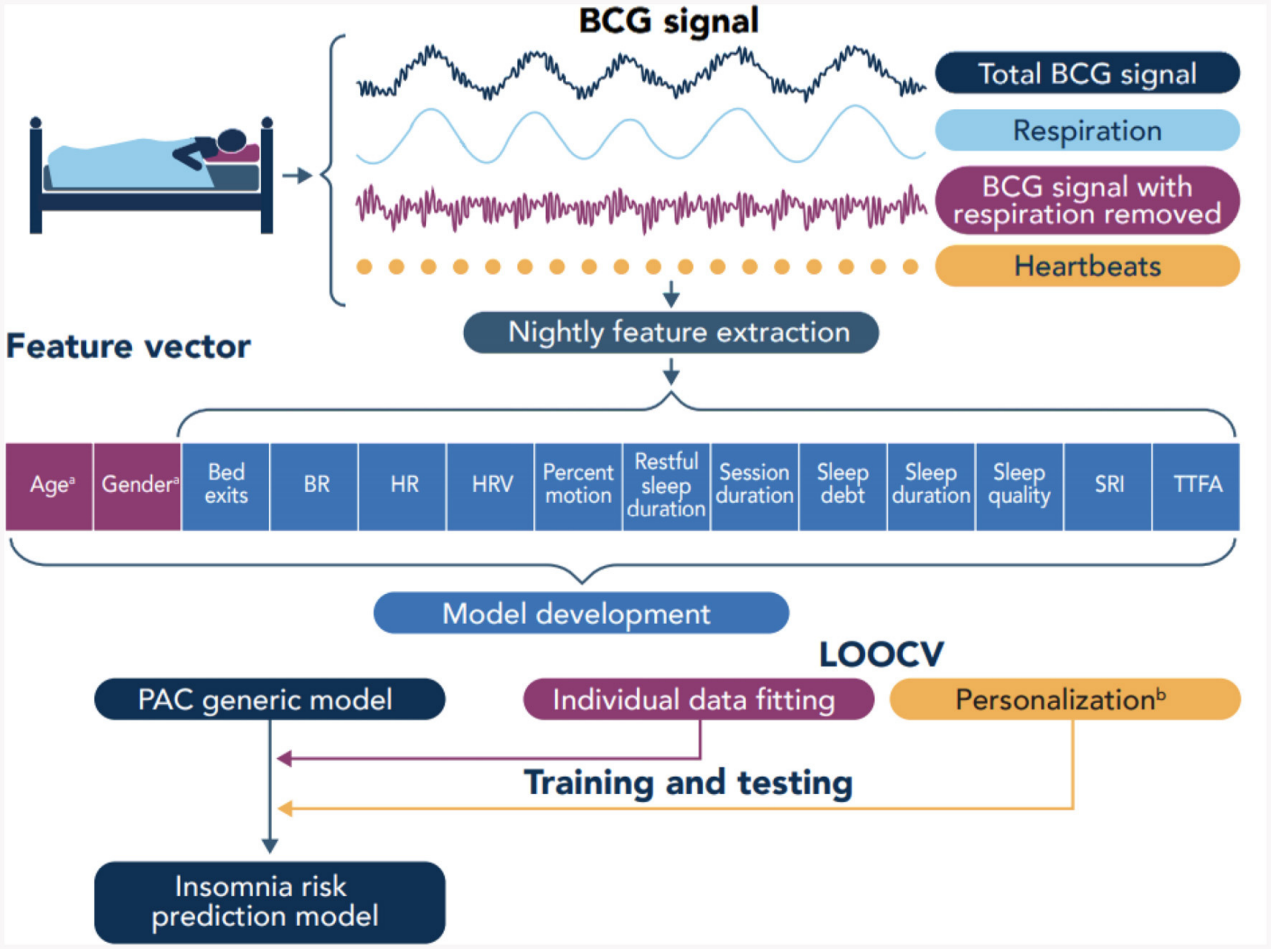
Overview of data collection and model development. BCG, Ballistocardiography; BR, Breathing rate; HR, Heart rate; HRV, Heart rate variability; SRI, Sleep regularity index; TTFA, Time to fall asleep; PAC, Passive agressive classifier; LOOCV, Leave-one-out cross-validation.

**Figure 4:**
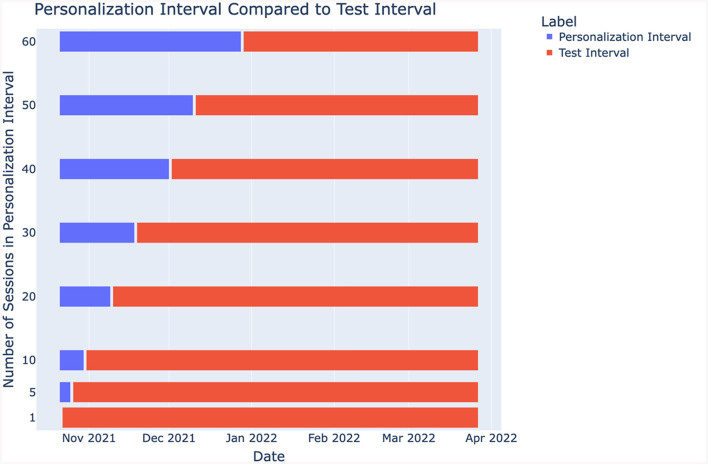
Personalization interval illustration.

We leveraged the transfer learning idea along with the leave-one-subject-out cross-validation (LOOCV) technique where the data from all but one subject are used to train a model which is tested on the data from the left-out subject. For each of the 1480 subjects in our dataset, we trained a generic passive-aggressive model (see Section 2.6) using the data from all other subjects, and we personalized the model using sleep session data from 1, 5, 10, 20, 30, 40, 50, and 60 days of the left-out subject (see also Figure 3). This is illustrated in Figure 4. The rest of the data from the left-out subject was used to evaluate the model performance.

### 2.6 Passive-aggressive learning

This is a binary online learning algorithm that makes predictions based on the error function's gradient, allowing it to adjust its predictions as new data are introduced (22). The passive-aggressive classifier updates its parameters incrementally and at the individual training sample level rather than at a batch (updates parameters after exposure to a fixed set of training samples) or epoch level (updates model parameters after a full pass over the entire training dataset). This makes the passive-aggressive approach ideal for the implementation of the personalized fine-tuning strategy described in the previous section. This classifier is passive in that it does not update its parameters when training samples are correctly classified and is aggressive in that it does update when incorrectly classifying training samples (22).

The passive-aggressive classifier has several hyper-parameters that can be adjusted to adjust its performance. In our implementation, we used a hinge loss which is zero for correct classifications and in case of misclassification increases proportionally to the distance from the sample to the decision boundary. The proportionality hyper-parameter controls the degree of aggressiveness in the updates to the decision boundary in the face of misclassification.

### 2.7 Performance metrics

We evaluated the performance of the personalized model using accuracy (Equation 1), precision (Equation 2), recall (Equation 3), and F1 score (Equation 4). In Equations 1–4, TP and TN represent the number of true positives and true negatives respectively. For each ISI threshold and personalization interval, we computed the average and standard deviation for each metric. As it is usually done with binary classifiers (23), we have also calculated the area under the receiving-operator curve (AUC) which characterizes the trade-off between true positive and false positive rate.


(1) $$\begin{array}{l} Accuracy=\frac{TP+TN}{TP+TN+FP+FN} \end{array}$$

(2) $$\begin{array}{l} Precision=\frac{TP}{TP+FP} \end{array}$$

(3) $$\begin{array}{l} Recall\left(TPR\right)=\frac{TP}{TP+FN} \end{array}$$

(4) $$\begin{array}{l} F1=\frac{2*Precision*Recall}{Precision+Recall}=\frac{2*TP}{2*TP+FP+FN} \end{array}$$

## 3 Results

The demographic information for the final dataset of 1,489 respondents is reported in Table 3. In addition, the mean ISI values per questionnaire are also reported.

**Table 3:** Respondent statistics & ISI scores.

**Respondents**	**1,489**
Male/Female/Other	669/811/9
Age	51.72 ± 12.77
ISI survey 1	9.65 ± 5.23
ISI survey 2	9.29 ± 5.36
ISI survey 3	9.06 ± 5.27
ISI survey 4	8.88 ± 5.29

**Figure 5:**
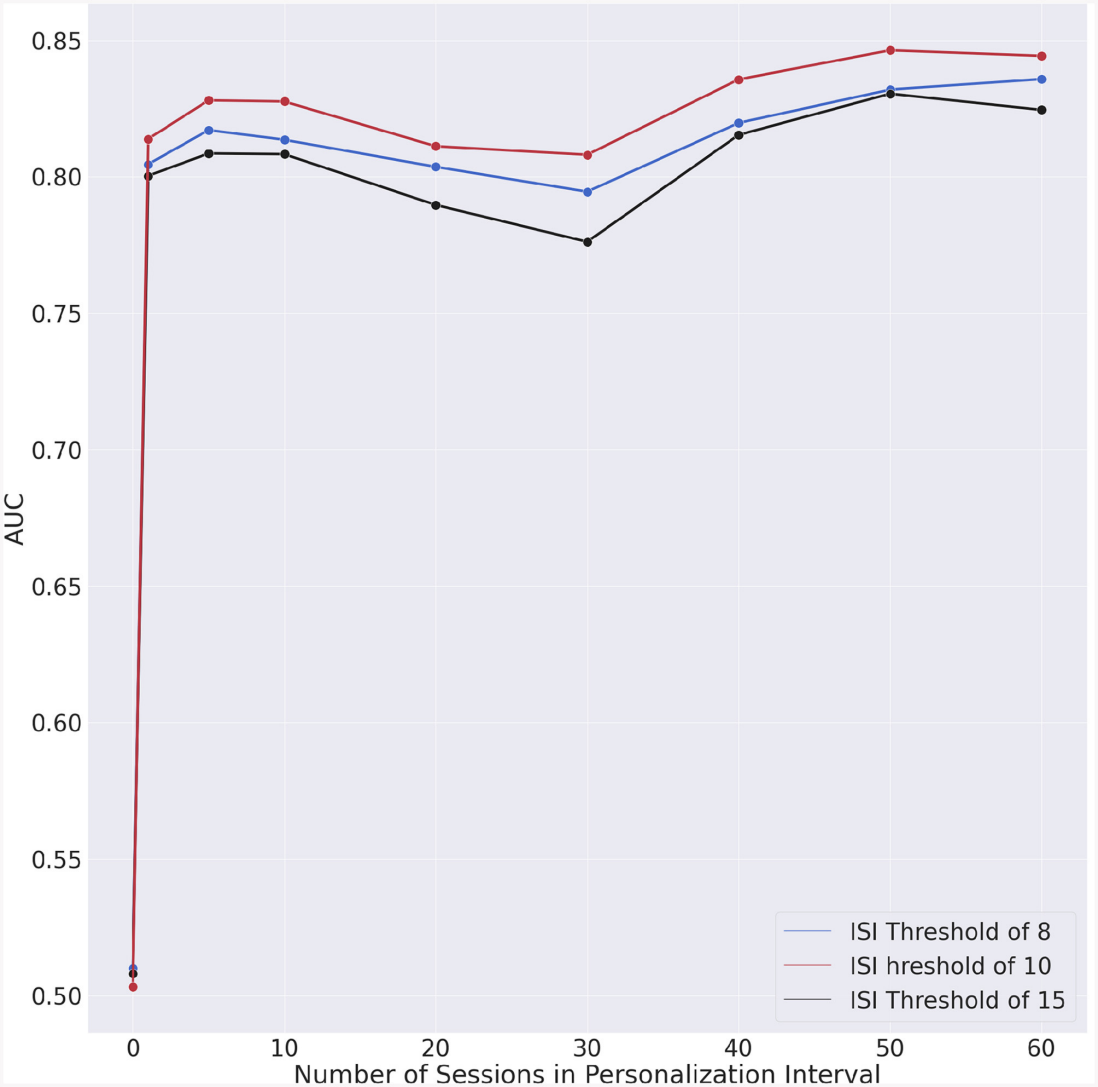
Area under the receiving-operator curves (AUC) vs. personalization interval for each ISI threshold.

The model's performance without personalization, i.e., the duration of the personalization interval is zero, serves as baseline for comparison. The metrics for all ISI thresholds and personalization interval are reported in Table 4. Figure 5 shows the mean AUC for each ISI threshold and personalization interval.

**Table 4:** Results for each ISI threshold and personalization interval duration.

**ISI thres**.	**Personalization**	**Accuracy**	**Precision**	**Recall**	**F1**	**AUC**
8	0	0.516 ± 0.376	0.491 ± 0.435	0.468 ± 0.461	0.596	0.510
	1	0.798 ± 0.244	0.560 ± 0.430	0.612 ± 0.458	0.831	0.805
	5	0.817 ± 0.249	0.541 ± 0.440	0.626 ± 0.473	0.848	0.817
	10	0.818 ± 0.260	0.536 ± 0.444	0.629 ± 0.477	0.849	0.814
	20	0.809 ± 0.282	0.522 ± 0.449	0.629 ± 0.480	0.840	0.804
	30	0.797 ± 0.304	0.514 ± 0.453	0.621 ± 0.483	0.830	0.795
	40	0.799 ± 0.306	0.512 ± 0.455	0.591 ± 0.489	0.830	0.820
	50	0.801 ± 0.309	0.504 ± 0.458	0.577 ± 0.490	0.829	0.832
	60	0.798 ± 0.317	0.506 ± 0.460	0.576 ± 0.489	0.829	0.836
10	0	0.509 ± 0.377	0.309 ± 0.405	0.282 ± 0.412	0.438	0.503
	1	0.786 ± 0.246	0.405 ± 0.429	0.455 ± 0.465	0.761	0.814
	5	0.816 ± 0.251	0.390 ± 0.433	0.476 ± 0.488	0.798	0.828
	10	0.816 ± 0.262	0.383 ± 0.434	0.480 ± 0.493	0.797	0.828
	20	0.807 ± 0.285	0.370 ± 0.435	0.482 ± 0.497	0.787	0.811
	30	0.795 ± 0.306	0.358 ± 0.435	0.468 ± 0.497	0.773	0.808
	40	0.799 ± 0.306	0.358 ± 0.437	0.440 ± 0.493	0.774	0.836
	50	0.801 ± 0.307	0.356 ± 0.438	0.427 ± 0.490	0.773	0.846
	60	0.798 ± 0.316	0.360 ± 0.442	0.431 ± 0.489	0.767	0.844
15	0	0.727 ± 0.353	0.060 ± 0.197	0.053 ± 0.197	0.162	0.508
	1	0.858 ± 0.226	0.126 ± 0.278	0.174 ± 0.354	0.587	0.800
	5	0.868 ± 0.235	0.124 ± 0.276	0.193 ± 0.386	0.629	0.809
	10	0.867 ± 0.243	0.118 ± 0.273	0.195 ± 0.392	0.626	0.808
	20	0.860 ± 0.264	0.109 ± 0.267	0.197 ± 0.397	0.603	0.790
	30	0.850 ± 0.284	0.104 ± 0.265	0.184 ± 0.387	0.570	0.776
	40	0.851 ± 0.287	0.105 ± 0.268	0.156 ± 0.360	0.656	0.815
	50	0.860 ± 0.276	0.107 ± 0.270	0.153 ± 0.355	0.579	0.830
	60	0.858 ± 0.285	0.108 ± 0.274	0.150 ± 0.349	0.570	0.825

**Figure 6:**
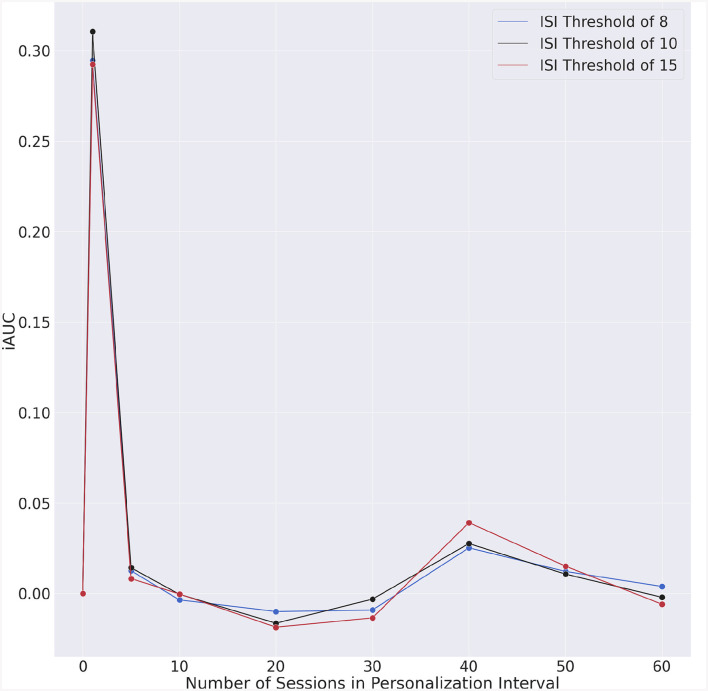
Incremental AUC vs. personalization interval for each ISI threshold.

The incremental AUC (iAUC) values for each ISI threshold are shown in Table 5 and Figure 6. These emphasize AUC improvements associated with the increase in the personalization interval. Improvements can already be observed when the personalization interval increases from 0 to 1 day, highlighting the immediate impact of incorporating even minimal personalized data into the model. Following the initial improvement, the iAUC values tend to diminish with some negative values recorded. This “diminishing return” trend suggests that personalization continues to contribute positively to the model's performance, the marginal gains decrease as more personalization data are incorporated.

**Table 5:** Incremental AUC.

**Personalization**	**8**	**10**	**15**
0	0.000	0.000	0.000
1	0.295	0.323	0.292
5	0.007	0.015	0.0151
10	0.001	–0.007	–0.004
20	–0.011	–0.013	–0.031
30	–0.009	0.003	0.006
40	0.032	0.021	0.022
50	0.015	0.003	0.014
60	0.002	0.002	0.006

The difference in iAUC for all possible pairs of ISI threshold was also statistically evaluated. The statistical significance of these differences are shown in Table 6.

**Table 6:** Statistical testing for iAUC values.

**iAUC**	**t-statistic**	***p*** **-value**
ISI 8–10	–0.035	0.972
ISI 8–15	0.022	0.983
ISI 10–15	0.056	0.956

## 4 Discussion

Our results suggest that significant accuracy improvement can be achieved by integrating longitudinal individual-specific data into an insomnia risk detection model. Such improvement may be due to the fact that insomnia symptoms impact sleep in an individualized manner. Indeed, the results across different personalization intervals and ISI thresholds show the difficulties of predicting insomnia risk; with near random results for a generalized model that does not account for individual differences. Even a modest amount of personalization was already sufficient to increase the AUC by 0.3 which represented a 60% improvement over the generic model which provided quasi-random results.

**Figure 7:**
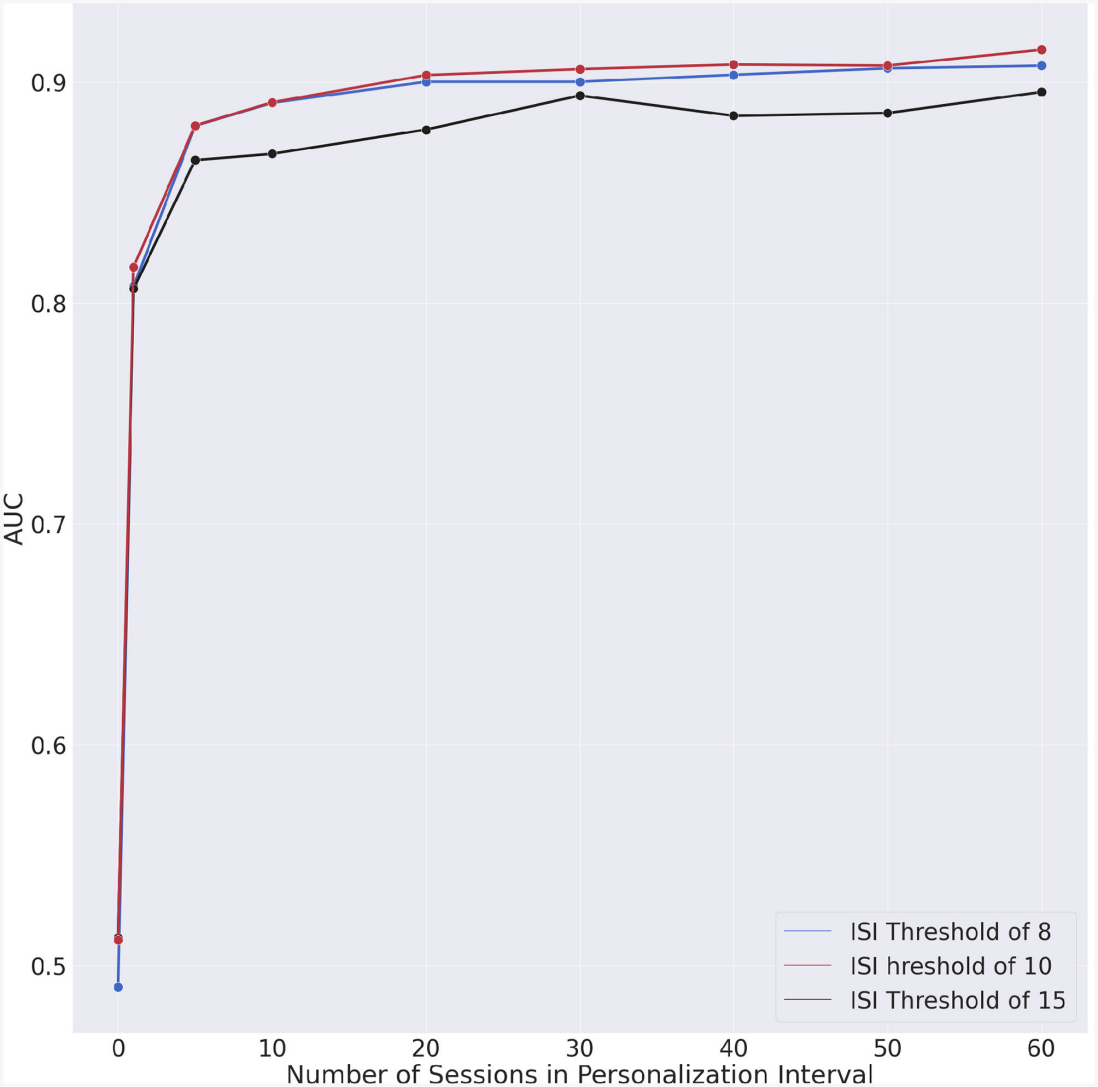
Experiment with chronologically randomized data. Incremental AUC vs. personalization interval for each ISI threshold.

We could also observe that the AUC (Figure 5) exhibits a slight degradation for approximately 30 days of personalization data. To understand whether this degradation is intrinsic to our model, we performed a test consisting in randomizing the data. In this manner, the chronologic information is no longer present in the data and if the degradation persists, then the specific machine learning algorithm would have caused that. The outcome of this experiment is shown in Figure 7. The fact that no AUC degradation can be observed in Figure 7 suggests that the decrease in AUC observed in Figure 5 may be due to the properties of the data. A plausible explanation for this degradation may be the proximity to the second questionnaire. However, no degradation could be observed for dates that are in the vicinity of the dates for the second or fourth questionnaires.

We considered three ISI thresholds in this research. The results in Table 4, Figures 5, 6 show similar trends for all considered thresholds. We performed a statistical comparison between the iAUC curves for all possible pairs of ISI thresholds (see Table 6). We did not find any statistically significant difference between any of the comparisons which suggests that there could be an equivalence in detecting insomnia risk by considering any of the three ISI thresholds we tested in this research. An appropriate threshold for insomnia risk is 10 which coincides with the choice by Oh et al. (7) and may better reflect the high prevalence of insomnia.

Our study has some limitations which are listed below.

- The population drawn from Sleep Number customers is not representative of the broader US population. This is reflected by the relatively older age of respondents reported in Table 3. Thus, the results reported in this research and the relevance of model personalization may not apply to the general population.
- The analysis reported in this research based on ISI threshold to reflect insomnia risk does not permit to identify a specific insomnia phenotype or the presence of comorbid sleep disorders such as sleep disordered breathing or restless leg syndrome. Comorbid conditions can influence the ISI and the features we consider in our model such as heart rate variability, heart rate, breathing rate, sleep quality, and sleep debt.
- Self-reporting insomnia and the electronic delivery cannot be considered as equivalent to diagnostics. Indeed, the respondent engagement and interaction with the electronic delivery method may be lower compared to in-clinic, and in-person questionnaire administration.
- The responses to multiple delivery of the same questionnaire even if done multiple weeks apart, may not necessarily be independent.
- While the smart bed has a pressure sensor for each sleeper on the bed, the nature of BCG is such that some minimal contribution of the signal produced by one bed user can reflect on that from the bed partner.

An opportunity to expand this research consists in considering insomnia phenotypes such as difficulty of falling asleep but normal sleep duration or normal sleep latency but difficulties of staying asleep. Indeed, the advantage of personalization may apply to insomnia phenotypes which could be easier to apply at a scale instead of individual level. An additional area for expansion is the prediction of insomnia over shorter intervals to enable detection of acute insomnia which if not treated early enough can convert into chronic insomnia.

The combination of longitudinally and unobtrusively acquired sleep data with personalized machine learning models constitutes a paradigm that may be generalized across sleep medicine from early detection, endotype, and phenotype identification to enable treatment optimization, and recovery monitoring. This research presents early encouraging results supporting that vision.

## Data availability statement

The datasets presented in this article are not readily available because the approved consent form prevent us from making the survey data publicly available. Requests to access the datasets should be directed to the corresponding author.

## Ethics statement

The studies involving humans were approved by Western Institutional Review Board WCG-IRB. The studies were conducted in accordance with the local legislation and institutional requirements. The participants provided their written informed consent to participate in this study.

## Author contributions

TW: Conceptualization, Data curation, Formal analysis, Methodology, Software, Validation, Visualization, Writing – original draft. VC: Data curation, Software, Writing – review & editing. DG: Conceptualization, Data curation, Formal analysis, Investigation, Methodology, Software, Writing – review & editing. MA: Conceptualization, Investigation, Methodology, Supervision, Validation, Writing – original draft, Writing – review & editing. SB: Data curation, Methodology, Software, Writing – review & editing. SD: Conceptualization, Funding acquisition, Methodology, Project administration, Writing – review & editing. BG: Data curation, Formal analysis, Software, Writing – review & editing. FM: Funding acquisition, Project administration, Resources, Writing – review & editing. GG-M: Conceptualization, Investigation, Methodology, Project administration, Resources, Supervision, Writing – original draft, Writing – review & editing.
